# Event and Comment

**Published:** 1916-02

**Authors:** 


### DENTAL REGISTER

Vol. LXX FEBRUARY, 1916 No. 2

### EVENT AND COMMENT

ANOTHER GIFT TO THE EASTMAN DISPENSARY. The daughters of Frank Ritter, who established the Ritter Dental Manufacturing Co., have just agreed to equip the new infirmary with chairs, engines, etc., to the extent of $20,000, as a memorial to their father. This gift has been accepted by Mr. Eastman, who will add the same amount to his great benefaction. When a good thing gets started every one seems inclined to boost, and we know of no more worthy object than these ladies have selected for their benefaction. We are wondering if Mr. Eastman will continue to duplicate other gifts. If he does, there will be something doing in the future in Rochester. With the kind of men backing up this enterprise there will be deeds worthy of the cause, whether the money continues to come in or not; but when we have the happy combination of both brains and money we have a right to expect big results, and we are sure there will be no disappointment in this instance.

THE FORSYTHE LOVING CUP. The editor of *Oral Hygiene* is really in earnest about that memorial to the Forsythe brothers, and he is planning to get a gift of ten cents or more from every member of the dental profession, and it will be just like him to get a large number in the profession to take his view of the matter. It seems so little to do, but if every member of the profession should comply with Bro. Belcher's suggestion I am sure Mr. Forsythe would cherish the testimonial more than anything that could ever come to him. It's only a small matter, but it is really important that every dentist should respond. Do it now before you forget.

TO ADVERTISE OR NOT TO ADVERTISE. Under this heading an advertising agent presents in one of our widely read dental journals a plea for a kind of newspaper advertising, which, on its face, is an educational plea for the better care of our teeth, but which is so persistently kept up that it in time influences people to present themselves in the author's office for necessary service. The claim is that it helps the young dentist to a practice in a shorter time, and because of its altruistic form it is not to be compared with the unfair statements of the charlatan. We wonder if some people will ever get the true ideal of the professional man. From the very nature of the case the professional man can never adopt the ideal of the commercial man. The professional man is a servant of the people and it is his business to serve and not to exploit the people. No man can enter such a profession as ours and prosper in the best sense who keeps his eyes and trains his mind continually on what is sometimes called "the main chance." The dentist today who will adequately prepare himself to serve the people devotedly will not need to advertise for people to serve. The fact is that less than one-sixth of the people in this country have anything like necessary dental attention, and in our judgment not ten per cent. of that sixth portion have all the service they need or would be glad to pay for. If our statement is at all a reasonable estimate, why should there be an unseemly public scramble by the profession to secure patients? It is also a fact that if every person in these United States should determine to have his teeth put in comfortable repair there would not be enough dentists in the whole world to accomplish the task. But our advertising man will tell us that this is the very reason he advocates making known the necessity for dentistry, that the ignorant people may know of the benefits to be derived from dental service. Did any one ever know of an advertising dentist or physician spending his good money to educate the masses without making his identity so clear that he alone would receive the benefits? The usual form of dental advertisement calls attention always to the superior qualifications of the advertiser, or to the great advantage in prices which he alone is able to offer. The advertising game is always a self-centered propaganda, and that is one of the reasons why it is considered by the profession to be unethical. It is unethical because it is unfair. It is unfair to other members of the profession and it is always likely to be unfair to the public. After all, it is and always has been considered unprofessional to advertise our services in a competitive manner; and why, then, should we break with a tradition that binds us together as a brotherhood with the high and noble ideal of attaining the highest skill and knowledge that we may the better minister to human suffering? Our code of ethics is not old and obsolete. It is stronger and more binding to-day than when Hypocrates affirmed it two thousand years ago. It will live forever, because it is based on a fundamental truth that has been reaffirmed in every honorably intentioned dentist who has accepted and lived it conscientiously. It is one of the strongest factors in maintaining our professional equilibrium and why, then, should we not stop discussing it, since no one seems able to advance a satisfactory substitute? In our judgment, it has no essential connection with the bread and butter problem.

WHAT'S IN A NAME? It is said that "a rose by some other name would smell as sweet." All of which may be true, but are we sure that the *"Dental Items of Interest"* will be more esteemed than the journal edited by Ottolengui under the old title? It has been the experience of several of our journals to change their title, some two or three times, and in some cases the change in name has resulted in other incidental and commendable changes. As we contemplate this subject our thoughts turn to the literal significance of the names of our journals, and we are surprised to note that there is not that appropriateness as to contents that we should expect. If we take the name *Items of Interest,* and compare it with the kind of material published in that journal, we do not find its pages filled with items, but well considered theses on important matters concerning practice. In fact it does not give its pages at all to brief paragraphs on technical details. And if we take up the *Dental Review,* we are surprised to find its pages filled with society papers and their discussions. It would seem that a more appropriate name would be *The Dental Reporter. The Dental Summary* should contain a compilation of all the happenings in the dental world, but instead it prints society proceedings and news notes of deaths, burglaries, etc. The *Dental Digest* devotes most of its space to practice building and prosthetic dentistry. The DENTAL REGISTER makes no claim to registering all that transpires in dental literature or professional activities. *The Dental Cosmos* perhaps comes nearer to keeping faith with its name than most other dental journals, but even that excellent journal is not wholly consistent. We suppose after all that it is necessary that everything should have a name for purposes of identification by the postmaster and others who may be interested; but it would seem that our habit of eluding the responsibility of keeping faith with our christening might justify the practice of nick-naming.

